# Recent advances in understanding urethral lichen sclerosus

**DOI:** 10.12688/f1000research.7120.1

**Published:** 2016-01-22

**Authors:** Altaf Mangera, Nadir Osman, Christopher Chapple

**Affiliations:** 1 Department of Urology Research, Royal Hallamshire Hospital, Sheffield, UK

**Keywords:** lichen sclerosus, male lichen sclerosus, inflammatory dermatosis

## Abstract

Lichen sclerosus affecting the male genitalia is a poorly understood but potentially devastating condition. The natural history of the condition is beginning to be understood better with longer follow-up of patients. Recent long-term data suggest that circumcision for lichen sclerosus limited to the prepuce may not be curative as was once thought. In addition, surgical treatments should be followed up for longer periods as recurrences may occur after urethroplasty and perineal urethrostomy.

## Introduction

Lichen sclerosus (LS) is a chronic inflammatory dermatosis with anogenital and extragenital presentations, the former of which is the most common. The epidermis and dermis are affected, and the aetiology remains unknown. In this review, we will consider LS affecting the male urethra and penile skin only. In this area, the skin typically becomes thickened and appears white, and the normal tissue architecture may be destroyed, earning it the outmoded name “balanitis xerotica obliterans”. LS may affect the prepuce alone or may include the external urethral meatus or distal urethra, and in severe cases the entire urethra may be involved. This review will update the reader on the latest evidence regarding aetiology and management of the various stages of LS.

## Aetiology

The aetiology of LS remains unknown, but an autoimmune pathogenesis seems most likely. One study of vulval LS biopsies showed that oxidative stress at the cellular level may be responsible for the changes ^1^. A larger study of female LS samples reported antibodies to matrix protein I in 75% of patients ^2^. Another study, assessing 153 samples from men with and without LS, revealed a significant difference in serum extracellular matrix 1 antibody levels ^3^. It is not clear what role the antibodies have in the genesis of LS. Another study compared male and female genital LS and reported higher numbers of CD4 ^+^ cells and a lower percentage of FOXP3 ^+^ lymphocytes in male LS, but both were higher than controls ^4^. Interleukin-10-positive lymphocytes were lower in both compared with controls.

A clinical review of 329 patients found other autoimmune disorders to exist less commonly in men with LS ^5^. Men circumcised in childhood had the lowest risk, followed by men circumcised later. A viral aetiology has also been proposed as human papilloma virus was found in 50% of paediatric preputial tissue affected by LS by polymerase chain reaction ^6^. Urinary pooling in the prepuce is also postulated to lead to LS ^7^. It is also postulated that stricturing and obstruction of urine at the glans lead to the extravasation of urine into the glands of Littre, leading to the inflammation and spongiofibrosis seen in LS. The Koebner phenomenon, which occurs in damaged skin secondary to inflammation, also produces LS. So skin injury has been suggested as a possible trigger of LS in genetically predisposed people.

LS in men may be limited to the prepuce or also include the glans or may be more aggressive, and spongiofibrosis may extend to affect the entire anterior urethra, which is much more common in adult men than children. Cystoscopically, the mucosa looks white or grey. The chronic inflammation has also been associated with squamous cell carcinoma, although the evidence regarding this is debatable ^8^.

## Management

To the best of our knowledge, there are no reported studies in the literature prospectively following the natural history of LS. Therefore, most information is gained from reports of LS management. In children, LS may be managed with steroid application. In a double-blind placebo-controlled trial, it was found that steroids could be used to reverse early-stage LS; however, even boys with early LS were completely resistant to steroid treatment ^9^. Intralesional injection of triamcinolone has also been used to cause LS regression in mild cases but has a recurrence of 13% ^10^. Circumcision is reported to be definitive in 96% of boys ^11^. An interesting study of 99 patients having biopsies from different parts of the penis and urethra showed an interval of more than 10 years between circumcision and urethral involvement ^12^. Also, progression of LS from meatus to the bulbar urethra was suggested to occur over the course of many years. Therefore, reporting a circumcision to be “curative” in 96% is possibly erroneous as most reports do not follow patients up for this long.

In a retrospective study of adult men, those who had steroid treatment of earlier disease were less likely to have recurrence compared to those with more extensive disease who required urethroplasty ^13^. It is not clear from this study whether the men who required urethroplasty would have progressed to needing urethroplasty as their disease was more aggressive from the outset. It has also been reported that early and aggressive treatment of LS may help in preventing disease progression and recurrence, but again longer follow-up is required ^13^.

A recent study reported that 1 out of 5 boys who underwent circumcision subsequently required a meatotomy, and that almost all of these boys had previously not undergone a meatotomy ^14^. The use of topical steroids was also associated with a reduced need for later meatotomy; however, it may have been that only boys with mild LS received steroids and therefore were less likely to recur at the outset. After meatotomy in LS, approximately 1 out of 4 patients will restenose and require further surgery ^15^.

As it has been suggested that LS progresses from the meatus proximally toward the bulbar urethra over the course of many years ^12^, it is not known whether the natural history of the condition can be changed by meatotomy or excision and grafting of a distal stricture. In LS a more extensive meatotomy (which leads to a hypospadiac meatus), including the use of grafts to try to reduce recurrence rates, has been advocated ^16^. Malone has described a novel ventral and dorsal meatotomy with an inverted relaxing V incision with good results ^17^.

In cases of more progressive disease affecting the urethra more proximally, urethroplasty is advocated ^18^. Both one- and two-stage approaches for augmentation urethroplasty have been described, and the latter had a lower recurrence rate albeit with slightly shorter reported follow-up ^19^. In either case, a skin graft should not be used, because of the high risk of recurrence, and instead an oral mucosa graft is advocated ^20^. For single-stage repair, a urethral plate wider than 10 Fr is required and the disease should be mild. However, a recent study reported 90% success with the use of a one-stage urethroplasty with dorsal onlay oral mucosa grafts through a perineal incision ^21^. The main complication of this approach was meatal stenosis. Morey has suggested that, in his experience, an extended meatotomy may be able to circumvent this problem ^16, 22^.

In severe cases, when the patient is unable or unwilling to have major urethral reconstruction, a perineal urethrostomy is a reasonable option ^23^. Men will be required to sit to void but should retain sexual function. This is often considered to be the last line of management and has a risk of restenosis which requires further surgery ^24^. Small case series have been reported of recurrence of LS in the edges of the skin surrounding the perineal urethrostomy and this was treated with potent steroid cream ^25^. If this fails, further surgery is often required.

## Conclusions

LS is a little-understood condition of unknown aetiology. Observations have suggested an autoimmune pathology possibly related to chronic irritation of the urethra with urinary extravasation into the corpus spongiosum. If caught early, it has been shown to regress with steroids or potent anti-immune therapy. In the case of obstruction and irritation of the urethra, progression may occur. It is unclear whether early management of the obstruction arrests progression. Certainly patients presenting late may have involvement of the whole urethra.

Management involves a stepwise approach. The notion that circumcision is “curative” in more than 95% of patients with only foreskin involvement is probably inaccurate as recent data suggest a lag of more than 10 years for LS occurrence in the glans and urethra. Urethroplasty is feasible in these patients and should use oral mucosa. More data are required to determine whether there is a significant difference between the one- and two-stage approaches. Finally, a perineal urethrostomy may be appropriate for certain patients. With each of the management options, there is not one which can claim to “cure” the condition and therefore long-term follow-up is warranted.

## Abbreviation

LS, lichen sclerosus.
